# The Effectiveness of Hands-On Practice Combined with Multimedia Education in Oral Hygiene Instruction for Adolescent Orthodontic Patients

**DOI:** 10.3290/j.ohpd.c_2325

**Published:** 2025-11-26

**Authors:** Qingyun Lin, Yaxin Zheng, Yanqing Chen, Yizhen Lin

**Affiliations:** a Qingyun Lin Attending Doctor, Department of Orthodontics, Stomatology Hospital of Xiamen Medical College, Xiamen Key Laboratory of Stomatological Disease Diagnosis and Treatment, Xiamen City, Fujian Province, 361008 China. Study design, definition of intellectual content, literature research, clinical studies, experimental studies, data acquisition, data analysis, statistical analysis, manuscript preparation, manuscript editing and review.; b Yaxin Zheng Associate Chief Physician, Department of Orthodontics, Stomatology Hospital of Xiamen Medical College, Xiamen Key Laboratory of Stomatological Disease Diagnosis and Treatment, Xiamen City, Fujian Province, 361008 China. Clinical studies, experimental studies, and manuscript review.; c Yanqing Chen Associate Chief Physician, Department of Orthodontics, Stomatology Hospital of Xiamen Medical College, Xiamen Key Laboratory of Stomatological Disease Diagnosis and Treatment, Xiamen City, Fujian Province, 361008 China. Clinical studies, experimental studies.; d Yizhen Lin Associate Chief Physician, Department of Orthodontics, Stomatology Hospital of Xiamen Medical College, Xiamen Key Laboratory of Stomatological Disease Diagnosis and Treatment, Xiamen City, Fujian Province, 361008 China. Guarantor of the integrity of the entire study, study concepts, study design, definition of intellectual content.

**Keywords:** adolescents, hands-on learning, oral hygiene, orthodontics, multimedia, patient education

## Abstract

**Purpose:**

To evaluate the efficacy of a novel educational approach combining hands-on practice with multimedia resources for improving oral hygiene knowledge, attitudes, and behaviours among adolescent orthodontic patients.

**Methods and Materials:**

This randomised controlled trial involved 120 adolescent patients (aged 12–18) undergoing fixed orthodontic treatment at Hospital of Xiamen Medical College. Participants were randomly assigned to either the intervention group (n = 60), receiving comprehensive oral hygiene education combining hands-on practice with multimedia resources, or the control group (n = 60), receiving standard verbal instructions and a printed pamphlet. The intervention consisted of three monthly sessions over 12 weeks, followed by a 3-month follow-up. Primary outcomes included oral hygiene knowledge (20-item questionnaire), attitudes (OHIP-14 scale), and clinical parameters (plaque index and gingival index). Secondary outcomes comprised participant satisfaction, oral hygiene practice frequency, and multimedia resource usage.

**Results:**

The intervention group demonstrated significantly greater improvements across all primary outcomes. Oral hygiene knowledge scores increased from 12.3 ± 2.8 to 17.9 ± 1.8 at follow-up in the intervention group versus 12.1 ± 2.9 to 13.8 ± 2.5 in controls (P < 0.001). OHIP-14 scores decreased from 18.5 ± 5.7 to 10.8 ± 3.9 in the intervention group versus 18.3 ± 5.5 to 16.8 ± 5.0 in controls (P < 0.001). Plaque index reduced from 2.1 ± 0.5 to 1.0 ± 0.3 in the intervention group versus 2.0 ± 0.6 to 1.7 ± 0.5 in controls (P < 0.001). Gingival index decreased from 1.8 ± 0.4 to 0.9 ± 0.3 versus 1.7 ± 0.5 to 1.5 ± 0.4, respectively (P < 0.001). The intervention group showed 93% satisfaction, with significantly higher brushing frequency (89.5% vs 62.5%) and interdental cleaning frequency (77.2% vs 42.9%) than controls.

**Conclusion:**

The combination of hands-on practice and multimedia education significantly improved oral hygiene outcomes among adolescent orthodontic patients compared to traditional instruction methods.

**Clinical Recommendations:**

Orthodontic practices should consider implementing structured educational programmes combining hands-on training with digital tools, allocating time for practical sessions during appointments, and conducting regular follow-ups to reinforce proper techniques.

Orthodontic treatment has become increasingly common among adolescents, with estimates suggesting that up to 45% of children require some form of orthodontic intervention.^14^ While these treatments can significantly improve dental alignment and overall oral health, they also present unique challenges in maintaining proper oral hygiene. The presence of orthodontic appliances creates additional surfaces for plaque accumulation and complicates traditional cleaning methods, potentially increasing the risk of dental caries and periodontal disease.^15^ Recent research has highlighted that the oral microbiota and periodontal health in orthodontic patients require special attention, as orthodontic appliances serve as breeding grounds for microbial attachment, leading to qualitative and quantitative differences in oral microbiota activity.^13^

Adolescence is a critical period for establishing lifelong health behaviours, including oral hygiene practices. However, this age group often struggles with adherence to oral care regimens, particularly when faced with the additional complexities introduced by orthodontic treatment.^23^ The challenge for orthodontists and dental professionals lies not only in providing effective treatment but also in educating and motivating young patients to maintain optimal oral hygiene throughout their orthodontic journey. As Fleming and Pandis recently emphasised, the unpredictable nature of post-treatment changes necessitates a comprehensive approach to both active treatment and retention phases, with careful supervision and patient education being paramount.^5^

Traditional approaches to patient education in orthodontics have primarily relied on verbal instructions and printed materials. While these methods have shown some efficacy, their impact on long-term behaviour change has been limited.^10^ In recent years, there has been a growing interest in leveraging multimedia and interactive educational strategies to enhance patient understanding and compliance. Studies have demonstrated that multimedia interventions can significantly improve knowledge retention and skill acquisition in various healthcare settings.^18^

The concept of hands-on learning, rooted in experiential learning theory, has long been recognised as an effective pedagogical approach across various disciplines.^8^ In the context of oral health education, hands-on practice allows patients to develop tactile skills and muscle memory, which are crucial for effective plaque removal and maintenance of orthodontic appliances. When combined with multimedia resources, this approach can create a more engaging and comprehensive learning experience for adolescent patients. This is particularly important given recent evidence showing that inflammation and mechanical forces during orthodontic treatment can significantly affect bone remodelling, making proper oral hygiene essential for preventing adverse periodontal outcomes.^7^

Recent technological advancements have opened up new possibilities for integrating multimedia elements into patient education. Virtual reality simulations, interactive mobile applications, and high-quality instructional videos offer innovative ways to demonstrate proper oral hygiene techniques and reinforce key concepts.^23^ These tools can provide patients with on-demand access to information and visual aids, potentially increasing their engagement with educational content outside of clinical settings. The importance of such technological approaches is underscored by recent advances in 3D evaluation methods for assessing gingival recessions and margin changes, which highlight the need for precise patient education about maintaining gingival health during orthodontic treatment.^6^

Despite the promise of these technologies, there is a paucity of research examining the combined effect of hands-on practice and multimedia education specifically tailored for adolescent orthodontic patients. Previous studies have investigated either hands-on approaches or multimedia interventions separately, but few have explored the synergistic potential of integrating these methods.^1,11^ Furthermore, special considerations are needed for patients with reduced periodontium, as recent research has shown that orthodontic movements such as intrusion and extrusion require careful management and patient education to prevent further periodontal damage.^2^

The management of uneven gingival margins and aesthetic considerations during orthodontic treatment further emphasises the importance of comprehensive patient education. As Martin et al recently noted, achieving optimal smile aesthetics requires not only proper orthodontic mechanics but also excellent patient compliance with oral hygiene protocols.^12^ Additionally, for patients with a history of periodontal disease, supportive periodontal care during active orthodontic therapy becomes crucial, necessitating more intensive educational interventions.^19^

Furthermore, while short- to medium-term improvements in knowledge and skills have been documented, sustaining these changes throughout the duration of orthodontic treatment presents an ongoing challenge.^23^ While this 3-month follow-up provides valuable initial insights into the sustainability of intervention effects, longer-term studies are needed to assess the efficacy of educational interventions throughout the entire orthodontic treatment period.

## Study Hypothesis

We hypothesised that adolescent orthodontic patients receiving combined hands-on practice and multimedia education would demonstrate significantly greater improvements in oral hygiene knowledge, attitudes, and clinical parameters compared to those receiving standard verbal instructions.

## Study Aim

The primary aim of this study was to evaluate the effectiveness of this integrated educational approach on oral hygiene outcomes at 3 months post-intervention, focusing on knowledge retention, attitude changes, and clinical improvements in this crucial demographic.

### METHODS AND MATERIALS

#### Study Design and Participants

This study employed a randomised controlled trial design to evaluate the effectiveness of a combined hands-on practice and multimedia education approach for improving oral hygiene among adolescent orthodontic patients. The study was conducted at the Hospital of Xiamen Medical College, over a period of 18 months from January 2023 to June 2024. Ethical approval was obtained from the Institutional Review Board, and the study was registered in the Clinical Trials Registry.

A total of 120 adolescent patients undergoing fixed orthodontic treatment were recruited for the study. The inclusion criteria were: (1) aged between 12 and 18 years; (2) undergoing fixed orthodontic treatment for at least 6 months; (3) no systemic diseases or conditions that could affect oral hygiene practices; and (4) willingness to participate in the study with informed consent from both the patient and a parent or guardian. Exclusion criteria included: (1) presence of cognitive or physical impairments that could interfere with oral hygiene practices; (2) concurrent participation in other oral health education programmes; and (3) planned relocation or inability to attend follow-up sessions.

Sample size calculation was performed using G*Power software (version 3.1.9.4), based on a previous pilot study. Assuming a medium effect size (Cohen’s d = 0.5), an alpha level of 0.05, and a power of 0.80, a minimum sample size of 51 participants per group was required. To account for potential dropouts, we increased the sample size by 15%, resulting in 60 participants per group.

#### Randomisation and Blinding

Participants were randomly assigned to either the intervention group (n = 60) or the control group (n = 60) using a computer-generated random number sequence. The randomisation was stratified by age (12–15 years and 16–18 years) and gender to ensure balanced distribution. Allocation concealment was maintained using sequentially numbered, opaque, sealed envelopes prepared by a research assistant not involved in the study.

Due to the nature of the intervention, it was not possible to blind the participants or the educators to the group assignment. However, the dental hygienists who conducted the clinical assessments were blinded to the group allocation. Data analysis was performed by a statistician who was also blinded to the group assignment.

#### Intervention

The intervention group received a comprehensive oral hygiene education programme that combined hands-on practice sessions with multimedia educational resources. The programme consisted of three sessions over a 12-week period, with each session lasting approximately 60 min. The sessions were conducted at 4-week intervals (baseline, week 4, and week 8).

#### Session 1: Introduction and Baseline Assessment

The first session began with a thorough explanation of the study objectives and procedures. Participants completed baseline questionnaires assessing their oral hygiene knowledge and attitudes. Clinical measurements, including plaque index (PI) and gingival index (GI), were recorded by calibrated dental hygienists. Following this, participants were introduced to the multimedia educational platform, which included an interactive mobile application and a series of high-quality instructional videos.

The mobile application, developed specifically for this study, featured 3D models of teeth with various orthodontic appliances, allowing users to virtually practice cleaning techniques. The instructional videos, professionally produced in collaboration with experienced orthodontists, covered topics such as proper brushing techniques with braces, the use of interdental brushes and floss threaders, and the importance of maintaining good oral hygiene during orthodontic treatment.

Participants were guided through the use of the application and watched the first set of instructional videos. They were then provided with a standardised oral hygiene kit containing a manual orthodontic toothbrush, interdental brushes, floss threaders, and fluoride toothpaste.

#### Session 2: Hands-On Practice and Skill Development

The second session, conducted one month after the first, focused on hands-on practice of oral hygiene techniques. Participants were divided into small groups of 5–6, each supervised by a dental hygienist. Using typodont models with fixed orthodontic appliances, participants practised brushing techniques, including the Modified Bass technique and the use of interdental cleaning aids.

The session incorporated real-time feedback using plaque-disclosing solutions on the typodont models, allowing participants to visualise areas of plaque accumulation and improve their cleaning techniques. The dental hygienists provided individualised guidance and corrected any errors in technique.

Following the hands-on practice, participants engaged in a group discussion about the challenges they faced in maintaining oral hygiene with braces and shared strategies for overcoming these difficulties. They were then assigned specific modules in the mobile application to reinforce the skills learned during the session.

#### Session 3: Reinforcement and Problem-Solving

The final session, held one month after the second, aimed to reinforce learned skills and address any persistent challenges. Participants’ oral hygiene status was reassessed using plaque and gingival indices. Individual feedback was provided based on these assessments, with particular attention given to areas showing inadequate improvement.

A problem-solving workshop was conducted, where participants worked in pairs to identify common oral hygiene issues associated with orthodontic treatment and propose solutions. This peer-to-peer interaction was designed to enhance engagement and promote the development of self-efficacy in managing oral hygiene.

The session concluded with a review of all educational materials and a discussion on maintaining long-term oral hygiene habits. Participants were encouraged to continue using the mobile application and to refer to the instructional videos as needed throughout their orthodontic treatment.

#### Control Group

The control group received standard oral hygiene instruction typically provided to orthodontic patients. This consisted of a single 30-min session with a dental hygienist, who provided verbal instructions on brushing and flossing techniques with braces. Participants in the control group were given a printed pamphlet containing information on oral hygiene practices for orthodontic patients and the same standardised oral hygiene kit as the intervention group.

#### Outcome Measures

The primary outcomes of the study were:

**Oral hygiene knowledge:** Assessed using a validated 20-item questionnaire covering various aspects of oral hygiene practices specific to orthodontic patients. The questionnaire was administered at baseline, immediately post-intervention (12 weeks), and at a 3-month follow-up.
**Attitudes towards oral health:** Measured using the Oral Health Impact Profile for Orthodontic Patients (OHIP-14), a validated 14-item scale assessing the psychosocial impact of oral conditions. This was administered at the same time points as the knowledge questionnaire.
**Clinical parameters:** PI and GI were recorded by calibrated dental hygienists at baseline, 12 weeks, and 3-month follow-up. The Silness and Löe plaque index and the Löe and Silness gingival index were used for these assessments.

Secondary outcomes included:

**Participant satisfaction:** Evaluated using a custom-designed satisfaction questionnaire administered to the intervention group at the end of the programme.
**Frequency of oral hygiene practices:** Self-reported through a daily log maintained by participants throughout the study period.
**Usage of multimedia resources:** App usage data was automatically collected through backend analytics integrated into the mobile application, while video viewing statistics were tracked through embedded counters.

#### Data Collection and Analysis

All data were collected using standardised forms and entered into a secure, password-protected database. Double data entry was performed to minimise errors. Statistical analysis was conducted using SPSS software (version 26.0, IBM, Armonk, NY, USA).

Descriptive statistics were calculated for all variables. Continuous data were expressed as means and standard deviations, while categorical data were presented as frequencies and percentages. The normality of data distribution was assessed using the Shapiro–Wilk test.

For comparing outcomes between the intervention and control groups, independent t-tests or Mann–Whitney U tests were used for continuous variables, depending on data distribution. Chi-square tests were employed for categorical variables. Repeated measures ANOVA or Friedman’s test was used to analyse changes in outcomes over time within each group.

A mixed-effects model was applied to account for the repeated measures design and to handle missing data. The model included fixed effects for group, time, and group-by-time interaction, with participant as a random effect.

All statistical tests were two-tailed, and a P value <0.05 was considered statistically significant. Effect sizes were calculated using Cohen’s d for continuous outcomes and odds ratios for categorical outcomes.

#### Material Availability

The mobile application and instructional videos developed for this study are currently being prepared for public release. Healthcare practitioners interested in implementing similar interventions can contact the corresponding author for access to these materials or guidance on developing comparable resources.

#### Ethical Considerations

The study was conducted in accordance with the Declaration of Helsinki and Good Clinical Practice guidelines. Informed consent was obtained from all participants and their parents or guardians before enrolment. Participants were informed of their right to withdraw from the study at any time without affecting their orthodontic treatment. All personal information was kept confidential, and the data were anonymised for analysis.

At the conclusion of the study, participants in the control group were offered access to the multimedia educational resources to ensure equity in care.

### RESULTS

#### Participant Characteristics

A total of 120 adolescent orthodontic patients were enrolled in the study, with 60 participants randomly assigned to each group. During the course of the study, three participants from the intervention group and four from the control group were lost to follow-up, resulting in a final sample of 57 and 56 participants in the intervention and control groups, respectively. The overall retention rate was 94.2%.

The demographic and baseline characteristics of the participants are presented in Table 1. There were no significant differences between the two groups in terms of age, gender, duration of orthodontic treatment, or baseline oral hygiene measures, indicating successful randomisation.

**Table 1:** Baseline characteristics of study participants

Characteristic	Intervention group (n = 57)	Control group (n = 56)	P value
Age (years), mean ± SD	15.3 ± 1.8	15.5 ± 1.7	0.547
Gender, n (%)			0.861
Male	28 (49.1%)	27 (48.2%)	
Female	29 (50.9%)	29 (51.8%)	
Duration of orthodontic treatment (months), mean ± SD	14.2 ± 4.5	13.9 ± 4.3	0.703
Baseline plaque index, mean ± SD	2.1 ± 0.5	2.0 ± 0.6	0.328
Baseline gingival index, mean ± SD	1.8 ± 0.4	1.7 ± 0.5	0.247
Baseline knowledge score, mean ± SD	12.3 ± 2.8	12.1 ± 2.9	0.698
Baseline OHIP-14 score, mean ± SD	18.5 ± 5.7	18.3 ± 5.5	0.845


#### Primary Outcomes

##### Oral hygiene knowledge

**Fig 1:**
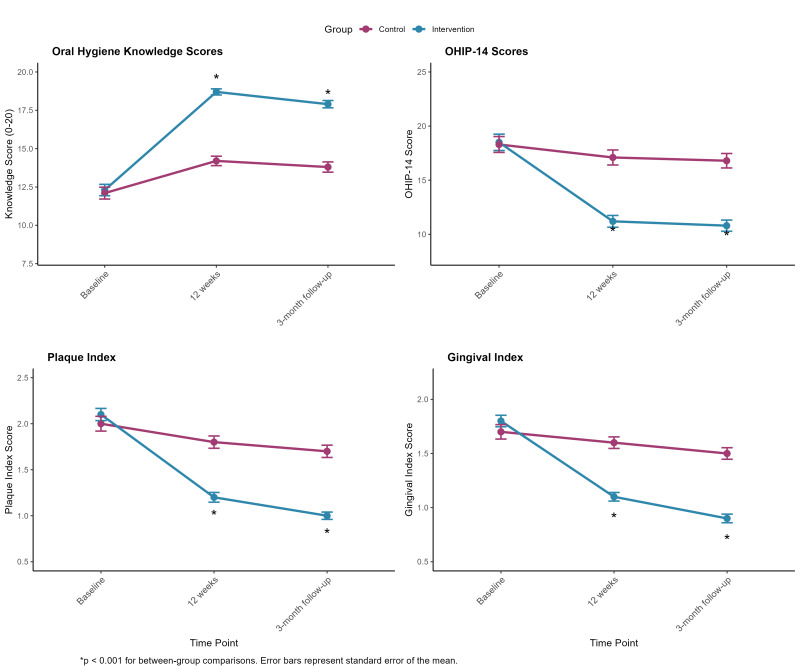
Changes in oral hygiene knowledge scores, OHIP-14 scores, and clinical parameters (plaque index and gingival index) over the study period. All measures showed significant improvements in the intervention group compared to the control group. Error bars represent the standard error of the mean.*P < 0.001 for between-group comparisons at 12 weeks and 3-month follow-up.

The intervention group demonstrated significantly greater improvements in oral hygiene knowledge compared to the control group over the study period. Table 2 presents the mean knowledge scores for both groups at baseline, 12 weeks (post-intervention), and 3-month follow-up. Figure 1 illustrates the changes in all primary outcomes over time.

**Table 2:** Changes in oral hygiene knowledge scores

Time point	Intervention group (n = 57)	Control group (n = 56)	Between-group difference (95% CI)	P value
Baseline	12.3 ± 2.8	12.1 ± 2.9	0.2 (–0.9 to 1.3)	0.698
12 weeks	18.7 ± 1.5	14.2 ± 2.3	4.5 (3.8 to 5.2)	<0.001
3-month follow-up	17.9 ± 1.8	13.8 ± 2.5	4.1 (3.3 to 4.9)	<0.001


Mixed-effects model analysis revealed a significant group-by-time interaction (F(2,222) = 47.62, P < 0.001), indicating that the change in knowledge scores over time differed between the two groups. The effect size (Cohen’s d) for the difference in knowledge scores between groups at the 3-month follow-up was 1.86, representing a large effect.

##### Attitudes towards oral health

Attitudes towards oral health, as measured by the OHIP-14, showed significant improvement in the intervention group compared to the control group. Lower scores on the OHIP-14 indicate better oral health-related quality of life. Table 3 presents the mean OHIP-14 scores for both groups at each time point.

**Table 3:** Changes in OHIP-14 scores

Time point	Intervention group (n = 57)	Control group (n = 56)	Between-group difference (95% CI)	P value
Baseline	18.5 ± 5.7	18.3 ± 5.5	0.2 (–1.9 to 2.3)	0.845
12 weeks	11.2 ± 4.1	17.1 ± 5.2	–5.9 (–7.6 to –4.2)	<0.001
3-month follow-up	10.8 ± 3.9	16.8 ± 5.0	–6.0 (–7.6 to –4.4)	<0.001


The mixed-effects model analysis showed a significant group-by-time interaction (F(2,222) = 31.15, P < 0.001). The effect size for the difference in OHIP-14 scores between groups at the 3-month follow-up was 1.39, indicating a large effect.

##### Clinical parameters

Both the PI and GI showed significant improvements in the intervention group compared to the control group. Table 4 presents the mean PI and GI scores for both groups at each time point. The visual representation of these changes is shown in Figure 1.

**Table 4:** Changes in plaque index (PI) and gingival index (GI)

Index	Time point	Intervention group (n = 57)	Control group (n = 56)	Between-group difference (95% CI)	P value
PI	Baseline	2.1 ± 0.5	2.0 ± 0.6	0.1 (–0.1 to 0.3)	0.328
	12 weeks	1.2 ± 0.4	1.8 ± 0.5	–0.6 (–0.8 to –0.4)	<0.001
	3-month follow-up	1.0 ± 0.3	1.7 ± 0.5	–0.7 (–0.9 to –0.5)	<0.001
GI	Baseline	1.8 ± 0.4	1.7 ± 0.5	0.1 (–0.1 to 0.3)	0.247
	12 weeks	1.1 ± 0.3	1.6 ± 0.4	–0.5 (–0.6 to –0.4)	<0.001
	3-month follow-up	0.9 ± 0.3	1.5 ± 0.4	–0.6 (–0.7 to –0.5)	<0.001


Mixed-effects model analyses revealed significant group-by-time interactions for both PI (F(2,222) = 53.78, P < 0.001) and GI (F(2,222) = 48.92, P < 0.001). The effect sizes for the differences between groups at the 3-month follow-up were 1.71 for PI and 1.63 for GI, both indicating large effects.

#### Secondary Outcomes

##### Participant satisfaction

Participants in the intervention group reported high levels of satisfaction with the combined hands-on and multimedia education programme. Table 5 presents the distribution of satisfaction scores.

**Table 5:** Participant satisfaction scores in the intervention group

Satisfaction level	Number of participants (%)
Very satisfied (5)	37 (64.9%)
Satisfied (4)	16 (28.1%)
Neutral (3)	3 (5.3%)
Dissatisfied (2)	1 (1.7%)
Very dissatisfied (1)	0 (0%)
	

The mean satisfaction score was 4.6 ± 0.5 on a 5-point Likert scale. Notably, 93% of participants rated the programme as either ‘satisfied’ or ‘very satisfied’.

##### Frequency of oral hygiene practices

Self-reported frequency of oral hygiene practices increased significantly in the intervention group compared to the control group. Table 6 presents the frequency of oral hygiene practices at the 3-month follow-up.

**Table 6:** Frequency of oral hygiene practices at 3-month follow-up

Practice	Intervention group (n = 57)	Control group (n = 56)	P value
Brushing at least twice daily	51 (89.5%)	35 (62.5%)	<0.001
Use of interdental cleaning aids at least once daily	44 (77.2%)	24 (42.9%)	<0.001
			

##### Usage of multimedia resources

Analysis of the mobile application usage data for the intervention group showed a high level of engagement. Table 7 presents the usage statistics of the multimedia resources.

**Table 7:** Usage of multimedia resources in the intervention group

Metric	Mean ± SD
Weekly app access frequency	5.3 ± 1.8
Total video views over 12 weeks	18.7 ± 4.2
Average time spent on app per week (min)	42.5 ± 15.3


##### Subgroup analysis

Exploratory subgroup analyses were conducted to examine the effects of age and gender on the primary outcomes. No significant interactions were found between these variables and the intervention effects, suggesting that the combined hands-on and multimedia education approach was equally effective across different age groups (12–15 years vs 16–18 years) and genders.

##### Adverse events

No adverse events related to the study interventions were reported during the course of the study.

### DISCUSSION

This randomised controlled trial investigated the effectiveness of a novel educational approach combining hands-on practice with multimedia resources for improving oral hygiene knowledge, attitudes, and behaviours among adolescent orthodontic patients. The results of this study demonstrate that this integrated approach is significantly more effective than traditional verbal instructions in enhancing various aspects of oral healthcare in this population.

#### Study Novelty

This study represents one of the first systematic investigations to combine hands-on practice with specifically designed multimedia education tools for adolescent orthodontic patients. The novelty of our approach lies in three key aspects: (1) the integration of tactile learning through typodont models with digital engagement via a custom-developed mobile application; (2) the structured three-session protocol that progressively builds skills and reinforces learning; and (3) the focus on the adolescent population, a group known for both high technology engagement and challenges with treatment compliance. Unlike previous studies that examined either digital tools or hands-on training in isolation, our integrated approach addresses multiple learning modalities simultaneously, potentially explaining the superior outcomes observed.

The substantial improvement in oral hygiene knowledge scores observed in the intervention group is particularly noteworthy. The mean knowledge score increased from 12.3 at baseline to 18.7 immediately post-intervention, with a sustained high score of 17.9 at the 3-month follow-up, representing a significant educational impact. This finding aligns with previous research suggesting that multimodal educational interventions can lead to better knowledge retention in healthcare settings.^18^ The combination of hands-on practice and multimedia resources likely contributed to this effect by engaging multiple learning modalities, thereby reinforcing key concepts and techniques. The interactive nature of the mobile application and the ability to revisit instructional videos may have played a crucial role in consolidating knowledge over time.

In contrast, the control group showed only modest improvements in knowledge scores, highlighting the limitations of traditional verbal instructions in conveying complex oral hygiene information. This disparity underscores the potential of innovative educational approaches in orthodontic care, particularly for adolescent patients who may be more receptive to technology-enhanced learning methods.^23^

The significant improvement in attitudes towards oral health, as measured by the OHIP-14, is another important finding of this study. The reduction in OHIP-14 scores from 18.5 at baseline to 10.8 at the 3-month follow-up in the intervention group indicates a substantial enhancement in oral health-related quality of life. This improvement may be attributed to several factors. Firstly, the hands-on practice sessions likely increased participants’ confidence in their ability to maintain proper oral hygiene with orthodontic appliances. This sense of self-efficacy is crucial in promoting positive attitudes towards oral healthcare.^3^ Secondly, the multimedia resources, particularly the mobile application, may have made oral hygiene routines more engaging and less burdensome for adolescents, leading to a more positive outlook on oral health maintenance.

The minimal change in OHIP-14 scores observed in the control group further emphasises the inadequacy of traditional instruction methods in addressing the psychosocial aspects of oral healthcare during orthodontic treatment. This finding is particularly relevant given the known challenges adolescents face in adapting to the demands of orthodontic care.^16^

Perhaps the most clinically relevant outcomes of this study are the significant improvements in PI and GI observed in the intervention group. The reduction in PI from 2.1 at baseline to 1.0 at the 3-month follow-up, and in GI from 1.8 to 0.9, represents a marked enhancement in oral hygiene status. These improvements are likely the result of improved plaque-removal techniques learned through hands-on practice and reinforced by multimedia resources. The ability to visualise proper brushing techniques through videos and practice them on typodont models may have contributed to more effective plaque control strategies.

The relatively minor changes in PI and GI observed in the control group highlight a critical gap in traditional orthodontic care. Despite receiving standard oral hygiene instructions, these participants did not achieve significant improvements in their clinical oral health status. This finding is consistent with previous studies that have reported suboptimal oral hygiene among orthodontic patients receiving conventional care.^15^ Our results suggest that more comprehensive and engaging educational approaches are necessary to effect meaningful changes in oral hygiene behaviours and outcomes.

The high level of participant satisfaction reported for the intervention programme is encouraging and supports the feasibility of implementing such approaches in clinical practice. The fact that 93% of participants rated the programme as either ‘satisfied’ or ‘very satisfied’ suggests that the combination of hands-on practice and multimedia education was well-received by adolescent patients. This positive reception is crucial for the success of any educational intervention, as it likely contributes to better engagement and adherence to oral hygiene practices.

The increased frequency of oral hygiene practices reported by the intervention group is another important outcome of this study. The finding that 89% of participants in the intervention group reported brushing their teeth at least twice daily at the 3-month follow-up, compared to 62% in the control group, suggests that the educational programme was effective in establishing better oral hygiene habits. Similarly, the higher rate of interdental cleaning aid use in the intervention group (78% vs 43%) indicates that the programme was successful in emphasising the importance of comprehensive oral hygiene practices for orthodontic patients.

The high engagement with the mobile application, as evidenced by the average of 5.3 weekly accesses and 18.7 video views over the 12-week intervention period, demonstrates the potential of digital tools in supporting ongoing oral health education. This level of interaction with educational content outside of clinical settings is particularly valuable in the context of orthodontic care, where consistent adherence to oral hygiene practices is crucial for treatment success and prevention of complications.^17^

The lack of significant interactions between age or gender and intervention effects in our subgroup analyses suggests that the combined hands-on and multimedia education approach is equally effective across different demographic groups within the adolescent population. This finding is important for the intervention’s generalisability and suggests it could be broadly implemented in orthodontic practices without extensive tailoring to specific age groups or genders.

Our findings contribute to the growing body of evidence supporting the use of technology-enhanced educational interventions in orthodontic care. Previous studies have demonstrated the potential of mobile applications and multimedia resources in improving patient compliance and oral health outcomes.^9, 23^ However, our study is unique in its integration of hands-on practice with digital tools, providing a more comprehensive approach to oral health education for orthodontic patients. This is particularly relevant in light of recent findings on the oral microbiota changes during orthodontic treatment, which emphasise the critical importance of maintaining excellent oral hygiene to prevent dysbiosis and associated complications.^13^

The success of this integrated approach can be understood within the framework of social cognitive theory.^4^ The hands-on practice sessions likely enhanced participants’ self-efficacy by providing mastery experiences, while the multimedia resources offered opportunities for observational learning through video demonstrations. The mobile application may have served as a tool for self-regulation, allowing patients to monitor their oral hygiene practices and receive ongoing guidance. This multi-faceted approach addresses various aspects of behaviour change, potentially explaining its superior effectiveness compared to traditional instruction methods.

The sustained improvements observed at the 3-month follow-up are particularly encouraging, as they suggest that the intervention may have short- to-medium-term effects on oral hygiene behaviours. This is crucial in the context of orthodontic treatment, which typically extends over several months or years. The ability to maintain good oral hygiene practices throughout the treatment period is essential for preventing complications such as white spot lesions, caries, and gingivitis, which can compromise treatment outcomes and overall oral health.^20^ These findings are especially relevant considering recent evidence on the importance of supportive periodontal care during active orthodontic therapy, particularly for patients with a history of periodontal disease.^19^

#### Clinical Recommendations

Based on our findings, we recommend that orthodontic practices:

Allocate dedicated time for hands-on training sessions during initial appointments and at regular intervals throughout treatment.
Provide patients with access to multimedia educational resources, preferably through mobile applications that allow for continuous engagement.
Conduct regular follow-up sessions to reinforce proper techniques and address emerging challenges.
Consider implementing a structured three-session protocol similar to our intervention for newly bonded patients.
Utilise plaque-disclosing agents during hands-on training to provide immediate visual feedback.
Encourage peer-to-peer learning and problem-solving activities to enhance engagement and self-efficacy.

#### Limitations

Several limitations of this study should be acknowledged. First, the three-month follow-up period, while providing valuable initial insights, is relatively short considering the typical duration of orthodontic treatment. Longer-term studies are needed to assess whether the observed improvements are maintained throughout the entire treatment period. Second, this was a single-centre study, which may limit the generalisability of findings to other settings or populations. Third, we did not conduct a formal cost-effectiveness analysis, which would be valuable for practices considering the implementation of similar programmes. Fourth, the inability to blind participants and educators to group assignment may have introduced some bias, although clinical assessors were blinded. Finally, while our sample size was adequate for detecting differences in primary outcomes, it may have been insufficient for detecting smaller but clinically meaningful differences in subgroup analyses.

#### Future Directions

Our findings also have implications for the broader field of patient education in healthcare. The success of this multimodal approach in improving both knowledge and behaviours suggests that similar strategies could be effective in other areas of healthcare where patient compliance and self-care are critical. For example, the combination of hands-on practice and multimedia education could be adapted for diabetes management, asthma care, or postoperative rehabilitation programmes.

From a practical standpoint, the implementation of such a comprehensive educational programme in orthodontic practice may require initial investments in technology and staff training. However, the potential benefits, including improved patient outcomes and reduced complications, could offset these costs in the long term. Moreover, the high levels of patient satisfaction and engagement observed in our study suggest that such programmes could enhance the overall patient experience, potentially leading to better treatment adherence and patient retention.

The use of a mobile application in this intervention aligns with the increasing digitalisation of healthcare and the growing preference for on-demand, personalised health information among younger patients.^21^ By leveraging familiar technologies, orthodontic practices can create more engaging and accessible educational experiences for their patients. This approach may be particularly valuable in reaching and motivating adolescent patients, who are often considered a challenging population in terms of treatment compliance.^22^

While our study focused on adolescent patients, future research could explore the efficacy of similar interventions in other age groups, such as adult orthodontic patients or younger children undergoing early orthodontic treatment. Additionally, investigating the long-term effects of such educational programmes beyond the 3-month follow-up period would provide valuable insights into the sustainability of improved oral hygiene practices over the entire course of orthodontic treatment.

One aspect that warrants further exploration is the potential for personalisation of the educational content based on individual patient needs and learning preferences. Future iterations of the mobile application could incorporate adaptive learning algorithms to tailor the educational experience to each patient’s progress and areas of difficulty. This personalised approach could further enhance the effectiveness of the intervention and improve patient engagement.

The positive results of this study also raise questions about the potential for expanding the scope of multimedia education in orthodontics beyond oral hygiene. Future research could explore the use of similar approaches for educating patients about dietary restrictions, management of orthodontic emergencies, or post-treatment retention protocols. By creating a comprehensive multimedia education platform, orthodontic practices could provide patients with a valuable resource throughout their treatment journey.

It is important to acknowledge that while the combined hands-on and multimedia education approach showed significant benefits, it may not be suitable or necessary for all patients. Some individuals may achieve adequate oral hygiene with traditional instruction methods, and the cost-effectiveness of implementing such comprehensive programmes for all patients needs to be carefully evaluated. Future studies could aim to identify specific patient characteristics or risk factors that predict the need for more intensive educational interventions.

The role of parental involvement in supporting adolescent patients’ oral hygiene practices is another area that deserves attention in future research. While our study focused on educating the patients themselves, incorporating elements that engage parents or caregivers in the educational process could potentially enhance the effectiveness of the intervention, particularly for younger adolescents.

In conclusion, this study demonstrates the significant benefits of combining hands-on practice with multimedia education for improving oral hygiene knowledge, attitudes, and behaviours among adolescent orthodontic patients. The integrated approach resulted in substantial improvements across all measured outcomes, including clinical parameters of oral health. These findings have important implications for orthodontic practice and patient education, suggesting that more comprehensive and engaging educational strategies are needed to optimise oral health outcomes in this population.

#### Acknowledgements

##### Ethical approval

All procedures performed in studies involving human participants were in accordance with the 1964 Helsinki declaration and its later amendments or comparable ethical standards. This study is approved by the Ethics Committee of Stomatology Hospital of Xiamen Medical College, with approval number KS20250507001, and written informed consent obtained.

##### Consent for publication

Informed consent was obtained from all individual participants included in the study.

##### Data availability

The simulation experiment data used to support the findings of this study are available from the corresponding author upon request.

##### Conflicts of interest

The authors declare that there are no conflicts of interest regarding the publication of this paper.

##### Funding

None.
